# The canine oral microbiome: variation in bacterial populations across different niches

**DOI:** 10.1186/s12866-020-1704-3

**Published:** 2020-02-28

**Authors:** Avika Ruparell, Taichi Inui, Ruth Staunton, Corrin Wallis, Oliver Deusch, Lucy J. Holcombe

**Affiliations:** WALTHAM Petcare Science Institute, Melton Mowbray, Leicestershire LE14 4RT UK

**Keywords:** Oral, Canine, Microbiome, Plaque, Buccal, Tongue, Saliva

## Abstract

**Background:**

Microbiota from different niches within the canine oral cavity were profiled and compared. Supragingival plaque and stimulated saliva, were collected alongside samples from the buccal and tongue dorsum mucosa, from 14 Labrador retrievers at three timepoints within a 1 month timeframe. The V3-V4 region of the 16S rRNA gene was sequenced via Illumina MiSeq.

**Results:**

Supragingival plaque microbiota had the highest bacterial diversity and the largest number of significant differences in individual taxa when compared to the other oral niches. Stimulated saliva exhibited the highest variability in microbial composition between dogs, yet the lowest bacterial diversity amongst all the niches. Overall, the bacteria of the buccal and tongue dorsum mucosa were most similar.

**Conclusions:**

The bacterial community profiles indicated three discrete oral niches: soft tissue surfaces (buccal and tongue dorsum mucosa), hard tissue surface (supragingival plaque) and saliva. The ability to distinguish the niches by their microbiota signature offers the potential for microbial biomarkers to be identified in each unique niche for diagnostic use.

## Background

The oral cavity represents an amalgamation of diverse, niche habitats all encompassed within a unique environment. As well as the compositional, structural and functional contribution of each component, the interaction of external influences such as food matter and care regimes, and microbial communities create an even more exclusive ecosystem.

To date, research into loci specific bacterial residents has focused primarily on hard rather than soft tissue surfaces [1, 2]. Microbial affinity for teeth is particularly robust, led via their preferential biofilm tendencies resulting in accumulations known in this context as plaque. Plaque build-up and its associated calcification (forming calculus) can be detrimental to periodontal health [3–6]. As a consequence, understanding the role microorganisms play in periodontal disease has influenced the bias towards analysis of subgingival plaque (plaque under the tooth’s gum line) [1, 7, 8]. Subgingival plaque sampling has indicated novelty in the canine microbiota compared to humans, with only 16.4% of taxa shared, and a low abundance of streptococcal species [1].

Supragingival plaque, which accumulates on teeth above the gum line, has also received some attention to understand relatedness to subgingival plaque and identify initial bacterial colonisers [9–12]. The potential microbial contribution of other niches within the canine oral cavity and associated risk to periodontal disease beyond subgingival plaque, however, remains unexplored. Given the extent and diversity of the ecosystem, a more universal understanding of the microbiome of the canine mouth beyond the teeth and gums, could not only deliver novel insights but also advance strategies for the prevention and/or treatment of periodontal disease.

Human studies have indicated subsets of microbiota are core to the health of the oral cavity of different individuals, variation in microbial profiles and diversity across different oral locations and stability in microbiota over time [13–17]. To the best of our knowledge, an extensive study of individual canine oral niches from a microbial perspective has not been conducted. The objective of this study was therefore to profile and compare the microbiota of different niches within the oral cavity of dogs. Ambition to deliver novel microbial insights creates the potential to identify niche-specific biomarkers of dental health; such developments could prove invaluable to the approach to canine periodontal disease in future.

## Results

### Samples and sequence quality

In total, 251 samples were collected from 14 Labrador retrievers: 84 supragingival plaque, 84 buccal mucosa, 42 tongue dorsum mucosa and 41 saliva. One saliva sample could not be collected due to lack of compliance of a dog during the first round of collections. Eight samples failed DNA amplification (2 buccal mucosa, 6 tongue dorsum mucosa).

Sequencing of the 16S rRNA V3-V4 region of the remaining 243 samples (84 supragingival plaque, 82 buccal mucosa, 36 tongue dorsum mucosa and 41 saliva) by Illumina MiSeq yielded a total of 44,931,668 forward and 44,931,668 reverse sequence reads from two runs. After processing through the bioinformatics pipeline, there were 3,741,324 assembled reads. Per sample, sequence reads ranged from 1 to 168,877 with specific median numbers of 13,159, 13,331, 13,372.5 and 10,264 for supragingival plaque, the buccal mucosa, the tongue dorsum mucosa and saliva samples respectively.

Five samples with counts under 1000 sequence reads, comprising 2 supragingival plaque, 1 tongue dorsum mucosa and 2 saliva samples were removed prior to statistical analysis. The total number of sequence reads remaining for the subsequent analysis was 3,739,825.

### Overall bacterial composition

The 3,739,825 assembled sequences were assigned to 223 operational taxonomic units (OTUs) following grouping of rare sequence reads into a separate group. The rare group accounted for 6.21% of the total sequence reads.

Assignment of taxonomy to each of the 223 OTUs resulted in 195 (87.4%) with ≥98% sequence identity to 16S sequences within the Silva database. The remaining 28 OTUs shared between 91.8 and 97.9% identity. Ninety of the 223 OTUs (41.4%) mapped to sequences of previously identified canine oral taxon (COT) [1] and another 17 (7.6%) mapped to sequences of previously identified feline oral taxon (FOT) [18]. The remaining 116 OTUs (52.0%) mapped to other taxa in the Silva database, of which 32 (14.3%) were designated with species level taxonomy.

A bacterial community composition analysis demonstrated that 202 of the 223 OTUs belonged to nine phyla: Proteobacteria (32.8%), Firmicutes (27.5%), Bacteroidetes (17.5%), Actinobacteria (4.5%), Fusobacteria (2.0%), Synergistetes (1.7%), Spirochaetes (0.7%), Tenericutes (0.5%) and Chlorobi (0.1%). The remaining 21 OTUs belonged to four candidate phyla: Saccharibacteria (3.8%), Absconditabacteria (1.6%), Gracilibacteria (0.6%) and WS6 (0.5%).

The 21 most abundant taxa (present at > 1%) across the study accounted for approximately 50% of the sequence reads (Table 1). An unclassified Pasteurellaceae sp. (OTU #21524) was the most abundant taxa representing 5.29% of the total number of sequence reads. An unclassified *Bergeyella* sp. (OTU# 4989), *Conchiformibius* sp. COT-286 (OTU# 10354) and *Porphyromonas cangingivalis* (OTU# 11671) were the next most abundant OTUs representing 3.74, 3.71 and 3.39% of the sequence reads, respectively. Eight other taxa each represented between 2.00 and 2.93% of the population. A further 18 OTUs represented between 1.00 and 1.99% of the population. The remaining 194 OTUs ranged in relative abundance from 0.0002 to 0.98%.

**Table 1:** The 21 most abundant operational taxonomic units

OTU	Assigned Taxonomy (Family/Genus/Species)	Percentage identity	Total Number of Sequence Reads	Proportion of total sequence reads (%)
21,524	unclassified Pasteurellaceae sp. [novel 1]	100.0	197,672	5.29
4989	unclassified *Bergeyella* sp. [novel 1]	100.0	139,841	3.74
10,354	*Conchiformibius* sp. COT-286	100.0	138,694	3.71
11,671	*Porphyromonas cangingivalis*	100.0	126,836	3.39
21,526	*Conchiformibius steedae* [novel 1]	99.77	109,642	2.93
30,042	unclassified *Escherichia-Shigella* sp. [novel 1]	100.0	100,307	2.68
1431	*Filifactor villosus*	100.0	99,658	2.66
25,622	unclassified *Frederiksenia* sp. [novel 1]	100.0	83,649	2.24
11,144	unclassified *Neisseria* sp. [novel 1]	100.0	79,361	2.12
31,443	unclassified *Proteocatella* sp. [novel 1]	100.0	75,675	2.02
1382	*Streptococcus minor*	100.0	75,553	2.02
10,651	*Moraxella* sp. FOT-350	100.0	74,779	2.00
20,323	*Neisseria weaveri*	100.0	72,670	1.94
12,643	unclassified *Capnocytophaga* sp. [novel]	100.0	68,468	1.83
31,690	unclassified *Porphyromonas* sp. [novel 1]	100.0	66,309	1.77
2753	Synergistales bacterium COT-178	100.0	62,658	1.68
26,919	Peptostreptococcaceae bacterium COT-005/004	100.0	60,786	1.63
30,902	Clostridiales bacterium FOT-072	100.0	60,116	1.61
9772	Peptostreptococcaceae bacterium COT-047	100.0	58,199	1.56
28,248	Peptostreptococcaceae bacterium COT-019	100.0	56,013	1.50
30,023	TM7 phylum sp. COT-305	100.0	53,472	1.43

Present at > 1% of total sequence reads across all the oral niches

### Comparison of bacterial composition across oral niches

**Fig. 1:**
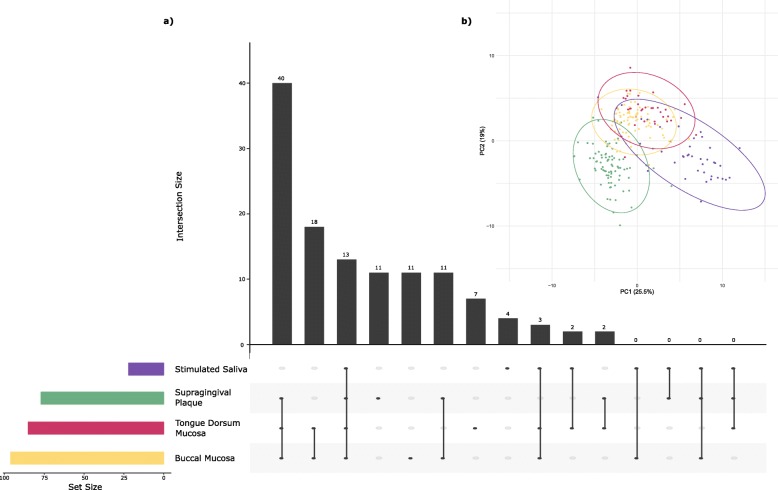
(**a**) UpSet plot based on presence/absence of operational taxonomic units where presence > 0.5% average OTU abundance mapped alongside (**b**) principal component scores with 95% confidence regions from analysis performed on the log_10_ proportions of operational taxonomic units identified in each of the oral niches: Buccal mucosa (yellow), supragingival plaque (green), saliva (purple) and tongue dorsum mucosa (magenta)

An UpSet plot was created to display the OTUs shared between the different canine oral niches (Fig. 1a). Common OTUs were calculated based on average OTU abundance being > 0.5% for each of the niches considered. The most OTUs (40) shown to be shared, were amongst samples of supragingival plaque, the buccal and tongue dorsum mucosa. At the other end of the spectrum, the following oral niche combinations were not found to share any of the study’s OTUs: saliva and the buccal mucosa; saliva and supragingival plaque; saliva, supragingival plaque and the buccal mucosa; and saliva, supragingival plaque and the tongue dorsum mucosa. All four niches were shown to share 13 OTUs.

Principal component analysis (PCA) was used to investigate differences across the samples and niches. The first component explained 25.5% and the second component 19% of the variability in the OTU log_10_ proportions (Fig. 1b). Little commonality was observed in the microbiota between all four oral niches, although the microbiota of the buccal and tongue dorsum mucosa were most similar. In addition, saliva from some dogs shared commonality in microbiota with the buccal and tongue dorsum mucosa. However, the majority of saliva samples formed their own cluster as did the supragingival plaque samples. Saliva samples indicated the highest variability of the four niches across dogs. To supplement these analyses, diet and gender were independently mapped onto the PCA analysis and no clustering by the three diets fed or sex was observed (data not shown).

**Fig. 2:**
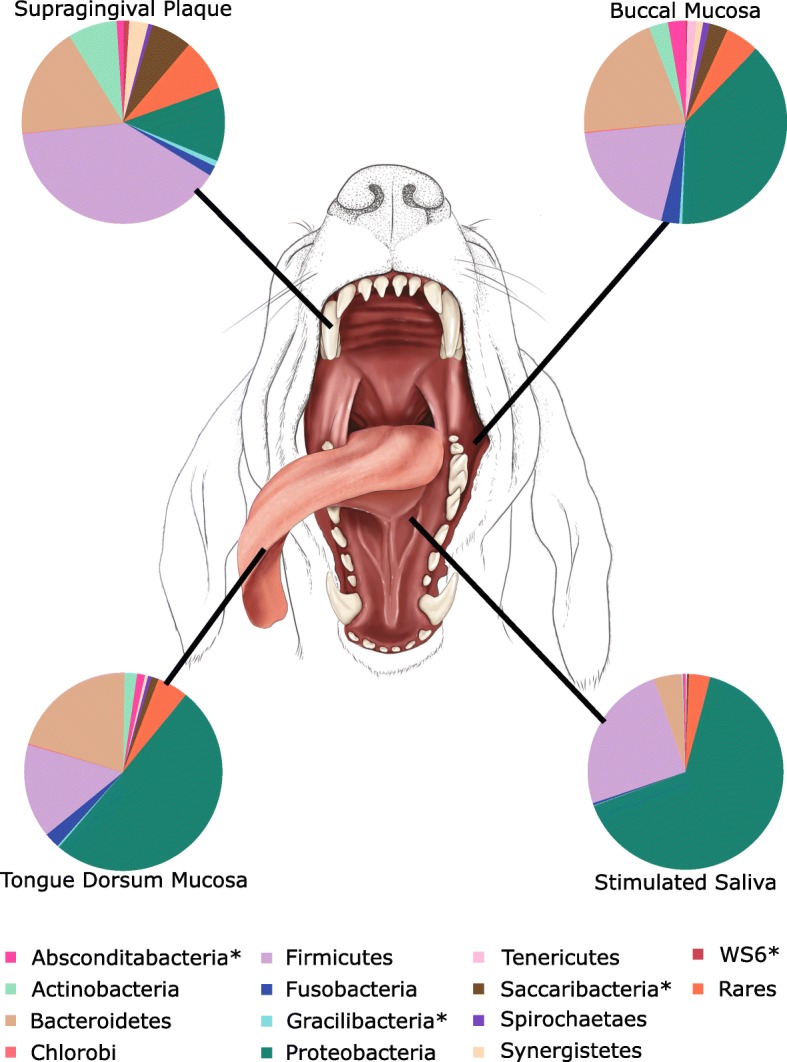
Average phylogenetic distribution of operational taxonomic units based on sequence reads across the canine oral niches. Asterisks indicate candidate phyla. Central image: © Emily McDougall Art

The phylogenetic distribution amongst the four niches is shown in Fig. 2. There was a consensus in the three most abundant phyla (Proteobacteria, Firmicutes and Bacteroidetes) across all the niches, although the ranking of these varied between the niches. Supragingival plaque was dominated by Firmicutes, Bacteroidetes and Proteobacteria, respectively. The buccal mucosa and tongue dorsum were both dominated by Proteobacteria, Bacteroidetes and Firmicutes, respectively. Saliva was dominated Proteobacteria, Firmicutes and Bacteroidetes, respectively. Supragingival plaque showed significantly higher proportions of Actinobacteria and candidate phyla Saccharibacteria than the other oral niches.

The OTU abundance lists also differed with each of the niches (Table 3 in Appendix). The most abundant taxa were *Filifactor villosus* (OTU# 1431) for supragingival plaque (5.16%), an unclassified Pasteurellaceae sp. [novel 1] (OTU# 21524) for the buccal mucosa (12.87%), *Conchiformibius* sp. COT-286 (OTU# 10354) for the tongue dorsum mucosa (17.27%) and an unclassified *Escherichia-Shigella* sp. [novel 1] (OTU# 30042) for saliva (20.46%).

**Fig. 3:**
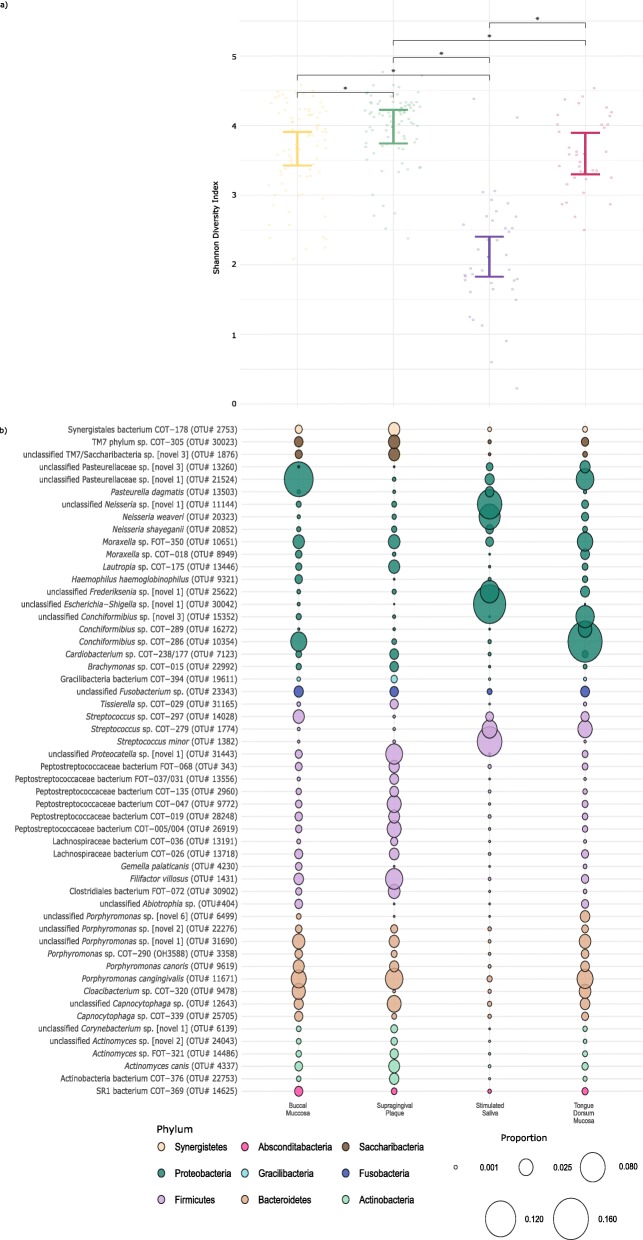
(**a**) Shannon diversity index with 95% confidence intervals (**b**) plotted against bacterial species indicating significant differences between the four niches within the dogs’ mouth. (**b**) indicates operational taxomomic units with abundance > 0.5% for at least one of the oral niches, where the size of the circles represent the proportion and the colours represent the phylum

Comparison of the OTUs identified within the different niches of the oral cavity sampled using univariate analysis showed supragingival plaque to be the most different to the other niches (Table 2). Comparisons among the buccal mucosa, tongue dorsum mucosa and saliva indicated fewer significant differences. The fewest number of significant differences were observed with the tongue and buccal mucosa comparison. Figure 3b illustrates the proportion of 54 OTUs, with proportions as percentages, across the four oral niches, indicating the largest, most contrasting observations. Complementing the phylum level findings in Fig. 2, supragingival plaque was dominated by several Firmicutes-associated taxa. These were largely characterised by multiple Peptostreptococcaceae sp. (0.010–0.028) and two Lachnospiraceae sp. (0.006–0.013). Further to that, and again consistent with the phyla analysis (Fig. 2), there were comparably fewer taxa represented by the other phyla, which demonstrated medium to low levels of abundance. Of these, most noteworthy were the abundance of the Bacteriodetes and Actinobacteria, with heavy representation of several *Porphyromonas* and *Actinomyces* species, respectively. In contrast, saliva was dominated by Proteobacteria, represented by the strong abundance of an unclassified *Escherichia-Shigella* sp. (0.150) and moderate abundance of two *Neisseria* sp. (0.064–0.086) and an unclassified *Frederiksenia* sp. (0.050). Saliva also demonstrated a variable abundance of three *Streptococcus* sp. (0.008–0.087) under the Firmicutes phylum, which were the next, and only remaining abundant taxa. Among this analysis, the buccal and tongue dorsum mucosa demonstrated most resemblance, with similar abundance of many of the bacterial taxa represented by the different phyla. The key differences observed here were among the Proteobacteria, where the most abundant bacterial taxa differed between the two niches. The tongue dorsum mucosa indicated a higher abundance of some *Conchiformibius* and *Moraxella* species compared to the buccal dorsum, while the buccal dorsum showed higher abundance of an unclassified Pasteurellaceae sp. [novel 1] in contrast to the tongue dorsum mucosa.

**Table 2:** Pairwise comparisons of the canine oral niches

	*p*-values < 0.05
Supragingival Plaque / Buccal Mucosa	125
Stimulated Saliva / Buccal Mucosa	86
Stimulated Saliva / Supragingival Plaque	104
Tongue Dorsum Mucosa / Buccal Mucosa	40
Tongue Dorsum Mucosa / Supragingival Plaque	92
Tongue Dorsum Mucosa / Stimulated Saliva	55

Indicates the number of operational taxonomic units (OTUs) that significantly differed. Numbers shown are out of 224 OTUs

### Diversity

The Shannon diversity index was significantly larger for supragingival plaque samples and significantly smaller for saliva samples compared to all other niches (*p* < 0.05) (Fig. 3a). Index values for samples from the buccal and tongue dorsum mucosa were not significantly different (*p >* 0.05).

### Gram-stain status and oxygen requirements

**Fig. 4:**
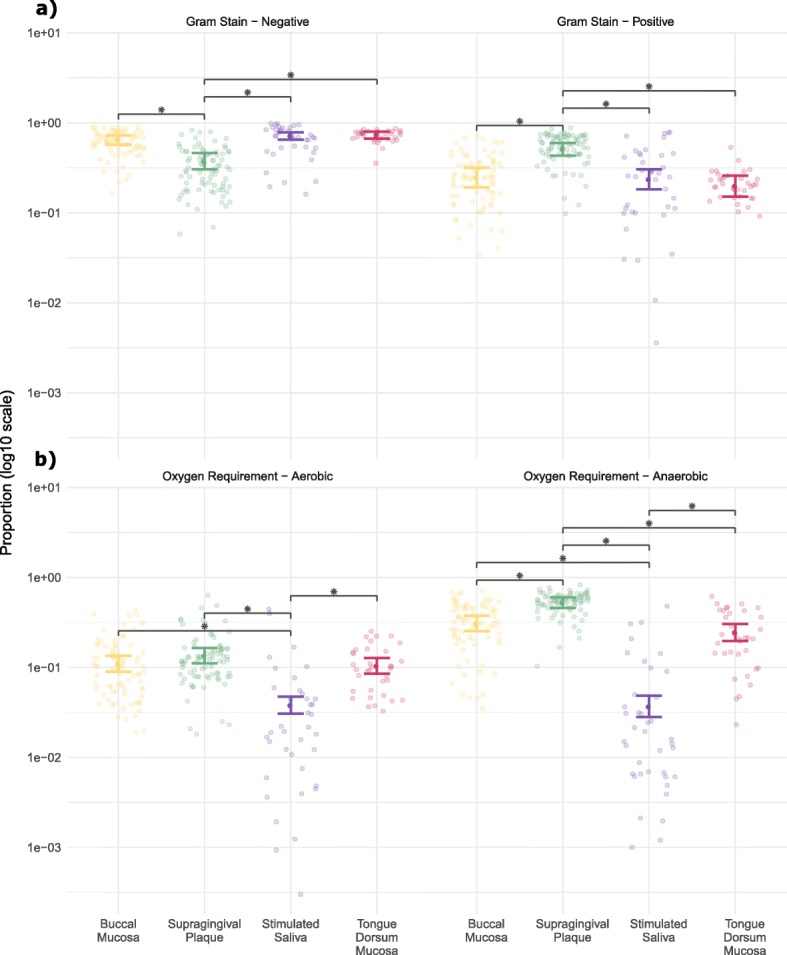
Proportions of bacterial species per sample discriminated by (**a**) Gram-stain status and (**b**) oxygen requirements. Coloured bars indicate mean proportions of OTUs with 95% confidence intervals across the canine oral niches: Buccal mucosa (yellow), supragingival plaque (green), saliva (purple) and tongue dorsum mucosa (magenta). Black bars and asterisks indicate niches with significant differences (all *p* < 0.001)

The probable Gram-stain status was determined by literature searches using the taxonomic identifiers applied to non-rare OTUs. Generalised linear mixed model (GLMM) analysis was then used to investigate differences between those proposed as Gram positive and Gram negative across the oral niches (Fig. 4a). Supragingival plaque samples had a significantly lower proportion of Gram negative OTUs and a significantly higher proportion of Gram positive OTUs than the buccal mucosa, saliva and the tongue dorsum mucosa (*p* < 0.001).

The oxygen requirements were also determined using the method described above for Gram stain status and GLMM used to investigate differences between aerobes and anaerobes across the oral niches (Fig. 4b). Saliva samples had a significantly lower proportion of aerobic and anaerobic OTUs than the buccal mucosa, the tongue dorsum mucosa and supragingival plaque (*p* < 0.001). This was likely due to the fact that saliva had lower bacterial diversity than the other oral niches (see above). Supragingival plaque samples also had a significantly higher proportion of anaerobic OTUs than the buccal and tongue dorsum mucosa (*p* < 0.001).

### Core microbiota

A core microbiota assessment was performed for each niche and then across all the niches. This was conducted as described by Turnbaugh et al. [19], using a detection threshold of ≥0.5% abundance across all the samples. For the individual niches, many OTUs were above the detection threshold across all the samples. For supragingival plaque, five OTUs including an unclassified *Bergeyella* sp. (OTU# 4989), an unclassified *Capnocytophaga* sp. (OTU# 12643), Lachnospiraceae bacterium COT-026 (OTU# 13718), Peptostreptococcaceae bacterium COT-047 (OTU# 9772) and an unclassified *Proteocatella* sp. (OTU# 31443) were detected across all 82 samples. Across all 82 buccal mucosa samples, four OTUs including an unclassified *Bergeyella* sp. (OTU# 4989), an unclassified *Capnocytophaga* sp. (OTU# 12643), *Porphyromonas cangingivalis* (OTU# 11671) and an unclassified *Porphyromonas* sp. (OTU# 31690) were identified. For the tongue dorsum mucosa, 9 OTUs could be identified ≥0.5% abundance across all 35 samples. The OTUs were an unclassified *Bergeyella* sp. (OTU# 4989), an unclassified *Frederiksenia* sp. (OTU# 25622), an unclassified Pasteurellaceae sp. (OTU# 25622), *Streptococcus* sp. COT-297 (OTU# 14028), *Moraxella* sp. FOT-350 (OTU# 10651), an unclassified *Fusobacterium* sp. (OTU# 23343), *Porphyromonas cangingivalis* (OTU# 11671), *Conchiformibius* sp. COT-286 (OTU# 10354) and an unclassified *Conchiformibius* sp. (OTU# 15352). For saliva, no OTUs were identified across the 41 saliva samples.

Across all the canine oral niches, no OTUs were identified across all samples at a proportion ≥ 0.5%. Three OTUs could be identified in 75% of the samples and 13 with 50% of the samples. The OTUs identified with 75% of the samples were an unclassified *Bergeyella* sp. (OTU# 4989), an unclassified *Capnocytophaga* sp. (OTU# 12643) and *Neisseria weaveri* (OTU# 20323). The additional OTUs identified with 50% of the samples were *Porphyromonas cangingivalis* (OTU# 11671), an unclassified *Fusobacterium* sp. (OTU# 23343), *Moraxella* sp. FOT-350 (OTU# 10651), an unclassified *Frederiksenia* sp. (OTU# 25622), Synergistales bacterium COT-178 (OTU# 2753), SR1 bacterium COT-369 (OTU #14625), an unclassified *Neisseria* sp. (OTU# 11144), *Capnocytophaga* sp. COT-339 (OTU# 25705), *Neisseria shayeganii* (OTU# 20852) and *Capnocytophaga* sp. COT-295 (OTU# 29107).

## Discussion

To our knowledge, this is the first study to provide a comprehensive microbial analysis of different oral niches in dog using a high throughput sequencing approach. Previous studies of the canine oral microbiota have concentrated on the sampling of subgingival plaque [1, 7, 8], supragingival plaque [10] or taken a composite sample of several oral niches [20, 21].

Assigning the bacterial taxa full taxonomy down to species level with or without COT or FOT identifiers has proven to be a key limitation in this study in terms of comparison of species to other work. Just over one third of the total OTUs were previously unidentified or novel taxa that could only be identified between the phylum and genus levels. This inability to provide the fullest possible taxonomy may be the consequence of sampling previously unexplored areas. At the time of characterization of the canine oral microbiome using subgingival plaque coupled with an advanced sequencing technology, 80% of the taxa identified were novel [1]. A constant effort to update and redefine the COT and FOT databases would be helpful in reducing any potential instances of this in the future.

An UpSet plot and PCA showed few of the microbiota sample profiles were shared between all four niches. Human studies suggest the tongue provides a reservoir for bacteria implicated in periodontal disease via supragingival dental plaque biofilms [22, 23]. The potential applicability of this to the canine model is questionable based on the current findings; the UpSet plot indicated 55 OTUs were shared between the two sample niches in the various combinations considered, while the PCA revealed very little overlap. However, another suggestion from the human field is that saliva reflects dislodgment of microbes from other surfaces [24]. This may hold some truth for dogs due to the commonality observed via PCA with saliva samples in relation to those of the soft tissue surfaces, although several of the Upset plot combinations established no OTUs to be shared.

A more prescriptive approach to analysing the OTUs and their assigned taxonomic identities, revealed some microbe-specific associations to the different niches considered. For the buccal and tongue dorsum mucosa, the bacterial signatures were difficult to distinguish, although a few key taxa under the Firmicutes phylum could be differentiated based on differing abundance. Overall, these findings are promising for the identification of biomarkers of dental health to aid improved diagnosis and/or treatment of canine periodontal disease. However, a thorough cross-sectional analysis that additionally considers disease samples would be needed to determine potential taxa-specific changes across the progressive stages of the disease.

While core microbiota have been shown for individual oral niches in the human literature, the genus level assignments generally differ to those observed here. On a global level, *Capnocytophaga*, *Neisseria*, *Porphyromonas* and *Fusobacterium* were observed amongst the core microbiota here, and also represent some of the genera which have been reported amongst humans [15, 16]. Furthermore, several species of these genera have been shown to be abundant amongst early (24 and 48 h) canine supragingival plaque biofilm [10].

Gram-stain status and oxygen requirements for taxa amongst human oral microbiota studies are not indicated. The observations with plaque here comprising significantly higher proportions of Gram positive, anaerobic and aerobic bacterial species than the other oral niches is interesting. While both hard and soft tissue surfaces are subject to biofilm formation, the observations suggest that despite supragingival plaque forming above the gum line, the nature of the biofilms is somewhat similar to canine subgingival plaque where the dominance of Gram-positive, aerobes in healthy samples has also been indicated [7]. The observation regarding anaerobes in supragingival plaque is, however, more conflicting, given the prior association of anaerobic microbes with disease samples, although in subgingival plaque [7]. This confounding observation is likely the consequence of differences in atmospheric oxygen levels between the two areas.

Microbial diversity varied across the canine oral niches. Supragingival plaque exhibited greatest species richness, saliva exhibited the least species richness, and the buccal and tongue dorsum mucosa were both in-between. While the findings for plaque are consistent, the observations for saliva provide contrast with parallel research in the human field. Several studies focusing on multiple oral surfaces report diversity parameters to be highest for both supragingival plaque and saliva [13–15, 25]. The lower diversity index values observed for the canine saliva population may be due to their mouths’ serving a multifunctional purpose. As well as chewing main meal diet and treats, this includes recreational chewing where residual saliva and the associated microbiota are lost from the oral cavity, thus diluting the remaining salivary microbiota when more saliva is stimulated. However, such behaviors also create opportunities for the acquisition of environmental microbes for dogs compared to humans.

## Conclusions

In summary, the oral niches can be distinguished into three groups based on their bacterial profiles: hard tissue surface (supragingival plaque), soft tissue surfaces (buccal and tongue dorsum mucosa) and fluid (saliva). The four niches demonstrated distinct taxa abundance and all except saliva core microbiota profiles. The variation in phylum composition, principal component and pairwise taxa analyses and microbial diversity parameters, however, were key in driving the divergence of the surfaces into the three groups. Not only does this enhance insights into microbial players in other areas within the canine oral cavity, but initiates the journey towards novel strategies to the prevention and/or treatment of periodontal disease.

## Methods

### Study design

The microbiota of the canine oral cavity were explored using dogs owned by the WALTHAM Petcare Science Institute and housed in accordance with conditions stipulated under the UK Animals (Scientific Procedures) Act 1986. Briefly, the dogs were pair housed in kennels designed to provide dogs free-access to a temperature controlled interior and an external pen at ambient temperature; dogs were provided with sleeping platforms at night. The dogs had access to environmentally enriched paddocks for group socialization and received lead walks and off-lead exercise opportunities during the day. Water was freely available at all times. The study was approved by the WALTHAM Animal Welfare and Ethical Review Body.

The study was a pilot, thus no power analysis was performed and fourteen Labrador retrievers were selected as a reasonable and convenient cohort size. The cohort consisted of 5 neutered males and 9 neutered females. The ages of the dogs at the beginning of the study were between 4.3 and 7.1 years (average age 4.9 years), and their bodyweights ranged between 22.4 and 30.8 kg.

The dogs used were all participants of another unrelated study which required them to be managed to prevent coprophagia. This allowed collection of supragingival plaque, food stimulated saliva and samples from the buccal and tongue dorsum mucosa without the risk of feacal contamination. Each sample type was collected on three occasions at least a week apart, over a period of 4 weeks. The dogs were fed one of three semi-purified diets (8.5% moisture, varying in the composition of methionine, as prescribed by another study taking place at the WALTHAM Petcare Science Institute. These were fed once a day to maintain bodyweight throughout and sampling was conducted at least 2.5 h after morning feeding of the diet. During the morning feed, 80% of the dog’s food allocation was offered and the remaining 20% was retained for treating during sample collection.

Sample collection was performed in the order in which the sampling for each niche is described. Supragingival plaque samples were collected from the outer buccal surfaces of tooth surfaces (maxillary third incisor, canine, third and fourth premolar and mandibular canine and fourth premolar on left and right side of mouth) using plastic microbiological loops (Scientific Laboratory Suppliers Ltd). Right and left side buccal mucosa were sampled by gentle scraping of these areas with a CytoSoft™ cytology brush (Medical Packaging Corporation). The posterior tongue dorsum mucosa was also sampled using a CytoSoft™ cytology brush. Plaque and buccal mucosa samples from the right and left sides of the mouth and tongue dorsum mucosa samples were placed into 2 ml collection tubes containing 1 ml Tris-EDTA (TE) buffer and immediately stored on ice. Left/right side plaque and buccal mucosa samples were processed separately. Stimulated, whole mouth saliva was collected using cotton wool swabs, and up to a 20% allocation of the dog’s diet, for treating and positive reinforcement. Saliva enriched cotton wool swabs were immediately collected into 50 ml centrifuge tubes (Corning Inc., USA) and stored on ice. Saliva was eluted by transferring the cotton wool swabs to Salivette collection tubes (Sarstedt, Germany) and centrifugation at 1000 rpm for 5 min. All samples were stored at − 80 °C prior to the extraction of DNA.

### DNA extraction

DNA was extracted from all samples using the Epicentre Masterpure Gram Positive DNA Purification Kit (Epicentre, USA) according to the manufacturer’s instructions with additional overnight lysis (see Davis et al. [7] for further details).

### Amplification of 16S rDNA

The variable V3-V4 regions of the 16S rDNA gene were amplified from the plaque DNA extractions. The universal bacterial primers to the 16S rDNA gene, 319F and 806R, each modified with a linker sequence, index sequence and heterogencity spacer as per Fadrosh et al. [26], were used for PCR amplification. The PCR mixtures (50 µL) contained 25 µL Phusion® High-Fidelity PCR Master Mix with HF Buffer (MO531, New England Biolabs, UK), 5 µL of each primer (1 µM), 10 µL template DNA, 3.5 µL nuclease free water and 1.5 µL DMSO, prepared in a 96-well format. The PCR cycling conditions consisted of an initial denaturation step at 98 °C (30 s), followed by 30 cycles of 98 °C (15 s), 58 °C (15 s) and 72 °C (15 s) and a final elongation at 72 °C (60 s). Successful amplification was confirmed through electrophoresis of the PCR products on 1.5% agarose gels.

### Library preparations and sequencing

Library preparation and sequencing were carried out by Eurofins Genomics, Germany. The 16S amplicons were pre-quantified using the Quant-iT™ PicoGreen® dsDNA Assay Kit (Invitrogen, UK). The diluted amplicons were then quantified using the Fragment Analyzer (Advanced Analytical Technologies, Inc.), then pooled into groups of 121/122 samples. The library pools were gel-sized prior to sequencing on a MiSeq (Illumina) with v3 chemistry, 2x300bp run modus.

### Sequence data processing

Forward and reverse reads were assembled into a contiguous sequences spanning the entire V3-V4 regions using *FLASH* assembler [27]. Tags were removed using *TagCleaner* [28] and sequences were demultiplexed in *QIIME* using split_libraries_fastq.py. Chimeric sequences were removed using userarch61 [29]. Sequences were clustered at > 98% identity using *uclust* [30] to generate OTUs and the most abundant sequences were chosen as cluster representatives. These were annotated with blastall 2.2.25 [31], which also contained canine and feline oral microbiome sequences previously published by the authors Dewhirst et al. and Pruesse et al. [1, 18, 32]. For further details please see Additional file 1.

### Statistical analysis

OTUs were combined in a single group of “rare” taxa if either they were present in each of the oral niches at an average proportion below 0.05% or were present in less than two samples. The 0.05% cut-off was selected based on statistical analysis of data from mock communities [7]. Samples with a total count of less than 1000 were excluded from analysis.

OTUs were analysed using binomial generalised linear mixed models (GLMM) with a logit link, using the count of an OTU out of the total number of sequences in a sample. Niche was included as the fixed effect and animal and sampling day as random effects. Left/right side plaque and buccal mucosa samples were considered as replicates in the analyses, as there was no hypothesis for a meaningful effect of mouth side. Using these models, mean proportions with 95% confidence intervals are reported for each niche and odds ratios with 95% confidence intervals are reported for all pairwise niche comparisons. Permutation tests were utilised to allay distributional assumption concerns: For each OTU, niche was randomly permuted within each animal. Test statistics were extracted for all between niche comparisons and the permutation *p*-value was calculated as the proportion of test statistics from permuted data which were more extreme than the test statistic from the original data. The permutation *p*-values were then adjusted according to the false discovery method of Benjamini and Hochberg [33] to allow for the increased likelihood of false positives when analysing the 224 OTUs. For each sample, counts were summed within each phylum and the binomial generalised linear mixed model with permutation testing methodology was applied to each phylum. Additionally, OTUs were assigned gram stain and oxygen requirement status and counts were summed within each level of both. The GLMM with permutation testing methodology was applied to each gram stain level and to each oxygen requirement level.

Multi-group PCA was performed on the log_10_ proportions, with dog as the grouping variable, to determine if clustering of samples was apparent. Ellipses representing the 95% bivariate confidence region for PC1 and PC2 were calculated for each niche and included on the PCA score plot [34].

Shannon diversity index [35] was calculated for each sample and a linear mixed model was used to analyse the indices, with niche as the fixed effect and animal and sampling day as random effects. Means for each niche and differences between niches are reported with 95% confidence intervals.

Statistical analyses were performed in R v3.3.3 [36] using *lme4* [37], *multcomp* [38], *ggplot2* [39], *snowfall* [40], *mixOmics* [41], *UpSetR* [42] and *ellipse* libraries [34].

### Supplementary information

**Additional file 1:** Sequence data processing.

## Data Availability

The datasets used and/or analysed during the current study are available from the corresponding author on reasonable request.

## Appendix

**Table 3:** The most abundant operational taxonomic units (OTUs) per niche: (a) supragingival plaque, (b) buccal mucosa, (c) tongue dorsum mucosa and (d) saliva

OTU	Assigned Taxonomy (Family/Genus/Species)	Percentage identity	Total Number of Sequence Reads	Proportion of total sequence reads (%)
(a) Supragingival plaque				
1431	*Filifactor villosus*	100.0	77,015	5.16
11,671	*Porphyromonas cangingivalis*	100.0	72,538	4.86
31,443	unclassified *Proteocatella* sp. [novel 1]	100.0	63,923	4.28
4989	unclassified *Bergeyella* sp. [novel 1]	100.0	61,339	4.11
26,919	Peptostreptococcaceae bacterium COT-005/004	100.0	50,964	3.41
30,902	Clostridiales bacterium FOT-072	100.0	50,429	3.38
9772	Peptostreptococcaceae bacterium COT-047	100.0	48,193	3.23
2753	Synergistales bacterium COT-178	100.0	45,641	3.06
12,643	unclassified *Capnocytophaga* sp. [novel]	100.0	43,829	2.94
28,248	Peptostreptococcaceae bacterium COT-019	100.0	40,047	2.68
1876	unclassified TM7/Saccharibacteria sp. [novel 3]		35,634	2.39
30,023	TM7 phylum sp. COT-305	100.0	34,574	2.32
(b) Buccal mucosa				
21,524	unclassified Pasteurellaceae sp. [novel 1]	100.0	164,340	12.87
21,526	*Conchiformibius steedae* [novel 1]	99.77	105,564	8.26
10,354	*Conchiformibius* sp. COT-286	100.0	47,684	3.73
4989	unclassified *Bergeyella* sp. [novel 1]	100.0	45,849	3.59
11,671	*Porphyromonas cangingivalis*	100.0	37,199	2.91
9478	*Cloacibacterium* sp. COT-320	99.76	36,618	2.87
31,690	unclassified *Porphyromonas* sp. [novel 1]	100.0	30,684	2.40
14,028	*Streptococcus* sp. COT-297	100.0	30,222	2.37
12,584	unclassified *Moraxella* sp. [novel]	99.07	26,517	2.08
9619	*Porphyromonas canoris*	100.0	26,133	2.05
10,651	*Moraxella* sp. FOT-350	100.0	24,867	1.95
404	unclassified *Abiotrophia* sp. [novel]	100.0	24,011	1.88
25,705	*Capnocytophaga* sp. COT-339	100.0	18,703	1.47
1431	*Filifactor villosus*	100.0	18,253	1.43
(c) Tongue dorsum mucosa				
10,354	*Conchiformibius* sp. COT-286	100.0	86,824	17.27
15,352	unclassified *Conchiformibius* sp. [novel 3]	100.0	28,290	5.63
21,524	unclassified Pasteurellaceae [novel 1]	100.0	24,193	4.81
1774	*Streptococcus* sp. COT-279	100.0	21,251	4.23
4989	unclassified *Frederiksenia* [novel 1]	100.0	18,416	3.66
25,622	unclassified *Frederiksenia* [novel 1]	100.0	18,370	3.65
16,272	*Conchiformibius* sp. COT-289	100.0	17,915	3.56
10,651	*Moraxella* sp. FOT-350	100.0	17,208	3.42
11,671	*Porphyromonas cangingivalis*	100.0	15,897	3.16
(d) Saliva				
30,042	unclassified *Escherichia-Shigella* sp. [novel 1]	100.0	95,822	20.46
1382	*Streptococcus minor*	100.0	72,087	15.40
11,144	unclassified *Neisseria* sp. [novel 1]	100.0	62,356	13.32

Represent approximately 50% of the total respective sequence reads in each niche. (a) supragingival plaque, (b) buccal mucosa, (c) tongue dorsum mucosa and (d) saliva

## Abbreviations

COT: Canine oral taxon
FOT: Feline oral taxon
GLMM: Generalised linear mixed model
OTUs: Operational taxonomic units
PCA: Principal component analysis
