# Supplementary material for: The canine oral microbiome: variation in bacterial populations across different niches
Source: BMC Microbiol. 2020 Feb 28;20:42. doi: 10.1186/s12866-020-1704-3 (PMC7048056; doi:10.1186/s12866-020-1704-3)
Supplement: Supplementary file 1 — Additional file 1: Sequence data processing. [file 12866_2020_1704_MOESM1_ESM.docx]

**Sequence data processing – Further details**

A dual-indexing and assembly approach as described by Fadrosh *et al.* [26] was used to sequence the V3-V4 region of the 16S gene on Illumina’s MiSeq platform. Paired-end raw sequence data in FASTQ format was treated as follows: Sequences and 12-mer barcodes were separated using the *trimfq* function of *seqtk* (v 1.2-r94) (https://github.com/lh3/seqtk/blob/master/README.md). Forward and reverse reads were assembled using *FLASH* (v 1.2.10 ) [28] with minimum and maximum overlaps of 40 and 200, respectively, and a maximum mismatch of 1% in the overlapping region. Tags were removed using *TagCleaner* (v 0.16) [29] with the settings -nomatch 3 -tag5 GGACTACHVGGGTWTCTAAT -mm5 3 -tag3 CTGCTGCCTCCCGTAGGAGT -mm3 3. Sequences were split into samples using split_libraries_fastq.py (v 1.9.1) from QIIME [31] using a phred score cut-off of 30 (-q 29, representing 99.9% base call accuracy) and a barcode length of 24 (2 x 12). Chimeric sequences were removed using userarch61 with the setting “reference free” [30].

Sequences were then clustered using *QIIME* version 1.9.1 (*ibid.*), using pick_otus.py (v 1.9.1), which utilises *uclust* (v 1.2.22q) [30] to cluster sequences with >98% identity [31] into Operational Taxonomic Units (OTUs). *Uclust* was run with modified parameters, with gap opening penalty set to 2.0 and gap extension penalty set to 1.0 and –A flag to ensure optimum alignment [31]. For each OTU cluster the most abundant sequence was chosen as representative sequence using pick_rep_set.py (v 1.9.1) also from *QIIME*.

Relative abundance and distribution across samples was assessed for each OTU in order to separate noisy from consistent but rare OTUs (refer to statistical analysis section for detailed description). Representative sequences of all OTUs that passed the filtering criteria were searched against the Silva SSU database release 128 [33] using blastall (v2.2.25) [32]. If the alignment did not meet the cut-off criteria of ≥98% sequence identity and ≥98% query sequence coverage genus or higher level annotations were used.
